# Employees’ Trust in AI and Innovative Behavior: A JD-R Model Perspective

**DOI:** 10.3390/bs16030425

**Published:** 2026-03-16

**Authors:** Chao Liu, Qichen Liao, Junting Lu

**Affiliations:** School of Business, Shandong University, Weihai 264209, China; liuchao@sdu.edu.cn (C.L.); 202317244@mail.sdu.edu.cn (Q.L.)

**Keywords:** trust in AI, job autonomy, concentration of work-related flow, innovative behavior, job complexity

## Abstract

With the rapid advancement of technology, whether to cultivate employees’ trust in artificial intelligence (AI) has emerged as a practical issue that managers must address to drive innovation. In this study, we explore how employees’ trust in AI affects their innovative behavior drawing on Job Demands-Resources (JD-R) theory with job autonomy and concentration of work-related flow as parallel mediators, and job complexity as a boundary condition. Using two-wave survey (with a two-week interval) data from 254 participants and structural equation modeling, we find that employees’ trust in AI positively relates to innovative behavior and this relationship is fully mediated by job autonomy and concentration of work-related flow. Furthermore, job complexity negatively moderates the trust in AI-mediator links and weakens the indirect effect on innovation. Based on the findings that enrich the literature on trust in AI and extend its boundary conditions, this study advises managers to cultivate employees’ trust in AI, leverage the resource-gaining and demand-enabling pathways, and adopt differentiated strategies tailored to job complexity to maximize innovation-enhancing effects of trust in AI.

## 1. Introduction

The digital intelligence era has elevated employee innovation to a determinant of enterprises’ competitive advantage, while technology assumes an indispensable role in fostering, scaling, and sustaining employees’ innovative behavior within organizational contexts (Raina et al., 2026). The rapid advancement of artificial intelligence (AI) technology has spurred enterprises to actively incorporate AI into their operational practices (Filippelli et al., 2026; Rammer et al., 2022), with such integration acting as a critical enabler for the development of new technologies and the promotion of organizational innovation. Nevertheless, AI technology is not innovation in its own right. To effectively harness AI for promoting innovation, enterprises must prioritize employees—the primary practitioners of innovative behavior—who mediate the translation of technological capabilities into actual innovation. Employees’ perceptions of AI technology exert a notable impact on their relevant attitudes, cognitions, and behaviors (J. Li et al., 2019). Among them, the degree to which employees believe that AI is trustworthy (i.e., trust in AI, Höddinghaus et al., 2021) may affect their decisions to apply new AI technologies to solve work problems (Bedué & Fritzsche, 2022), thereby influencing their innovative behavior (Scott & Bruce, 1994). Therefore, whether and how employees’ trust in AI affects their innovative behavior has become an important issue that needs to be solved in the current enterprise management practice.

As AI develops and is increasingly applied, scholars have confirmed the important role of trust in AI. The current studies found that trust in AI can promote the willingness to interact with AI (Johnson & Grayson, 2005), disclose information with AI (Esmaeilzadeh, 2020) and collaborate with AI (Kong et al., 2023). Moreover, research on the link between AI trust and innovation has yet to achieve a fully systematic approach (He et al., 2025; Kong et al., 2025), and few targeted explorations have been conducted into the association between employees’ trust in AI and their innovative behaviors from the perspective of systematic theoretical frameworks. Nevertheless, existing evidence indicates that trust in AI facilitates employees’ adoption of AI (Bedué & Fritzsche, 2022), which in turn contributes to promoting innovation (Zeng et al., 2022). In addition, employees’ AI-related perceptions (e.g., AI awareness) significantly influence their innovative behavior (Liang et al., 2022). Based on the above-reviewed evidence, there may be a nontrivial relationship between trust in AI and innovative behavior. To fill this research gap in the existing literature, the present study aims to thoroughly examine whether employees’ trust in AI affects their innovative behavior and systematically explore the specific pathways through which this influence occurs

The Job Demands-Resources (JD-R) model posits that job characteristics can be categorized into job resources or job demands, with individual factors potentially influencing the process and outcomes of this categorization (Demerouti et al., 2001). Employees’ trust in AI reflects their positive recognition of AI’s functionalities and attributes: they not only perceive AI as an effective tool but also strive to acquire AI-related knowledge and skills (Kong et al., 2021; Liang et al., 2022). In contrast, distrust in AI indicates their skepticism toward AI’s positive impacts, making them less likely to incorporate AI into their work methods or engage in relevant learning endeavors. Therefore, based on the JD-R model, trust in AI is likely to influence how employees perceive job demands and job resources. Specifically, within the resource pathway of the JD-R model, employees who trust AI perceive it as a useful tool—one that enriches their job resources(e.g., more energy and time), enhances job autonomy, and thereby generates a resource-gaining effect (Demerouti et al., 2001). In contrast, along the demand pathway, AI-trusting employees are more likely to set challenging job demands (e.g., learning AI more proactively and exploring how to apply it to work processes) for themselves (Kong et al., 2021; Liang et al., 2022) and proactively respond with resources (Bakker et al., 2010), which in turn elevates their level of work-related flow and fosters a demand-enabling effect. Further, existing research has consistently demonstrated that both job autonomy (Bindl et al., 2019; Černe et al., 2017; Orth & Volmer, 2017; Purc & Laguna, 2019; Slåtten & Mehmetoglu, 2011) and concentration of work-related flow (Hoffman & Novak, 2009; Liu et al., 2023; Maqbool et al., 2019; Peng et al., 2024) positively predict employees’ innovative behavior. Thus, based on the JD-R model, this research intends to investigate the mediating effects of job autonomy and concentration of work-related flow experience on the link between employees’ trust in AI and innovative behavior.

Further, scholars have emphasized that future research should validate the role of trust in AI in specific contextual settings (Glikson & Woolley, 2020; Vanneste & Puranam, 2024). Jia et al. (2024) argue that job complexity serves as a critical contextual factor in human-AI interaction. Indeed, given the inherent limitations of current AI technology (Kong et al., 2023), AI struggles to accomplish non-routine, non-procedural, and non-coding tasks (Jia et al., 2024), rendering it ineffective in addressing complex work demands. In other words, even if employees engaged in high-complexity work trust AI, they can barely leverage AI to tackle complex job tasks. Accordingly, this study argues that it is necessary to further examine the important contextual role of job complexity when trust in AI exerts its influence on employees.

This study makes three primary theoretical contributions. First, this study expands the theoretical perspective and outcome scope of trust in AI research by figuring out how employees’ trust in AI reshapes cognition and attitudes to influence innovative behavior, filling the gap regarding trust in AI’s positive effects and providing a theoretically rooted analytical framework in contrast to current research which lacks a systematic theoretical framework to some extent. Second, this study uncovers the “black box” of the mechanism linking trust in AI to innovative behavior by identifying job autonomy and concentration of work-related flow as parallel mediators, revealing dual pathways—a “resource-gaining effect” (enhancing job autonomy) and a “demand-enabling effect” (deepening concentration of work-related flow)—that enrich mechanistic understanding of AI perception and employee behavior. Third, we take job complexity into account by showing that employees with different levels of job complexity differ in their cognition of job demands and resources, as well as in their innovative behavior outcomes when they trust in AI. By integrating individual perception and work context, we enhance the contextual adaptability of this study, thereby broadening the research boundaries of trust in AI and providing implications for future research on boundary conditions in this field.

## 2. Theoretical Background and Hypotheses

### 2.1. Job Demands-Resources Model

The JD-R model exhibits notable relevance to the research on the impact of employees’ trust in AI on their workplace behaviors. This theoretical framework facilitates an explanation of how employees’ trust in AI reshapes the configuration of job demands and job resources, as well as the indirect effects of such changes on individual behavioral outcomes.

At its theoretical core, the JD-R model posits that all job characteristics can be dichotomized into two fundamental, mutually influential constructs: job demands and job resources (Demerouti et al., 2001). Job demands are defined as those physical, psychological, social, or organizational aspects of the job that require sustained physical and psychological effort, thereby entailing associated physiological or psychological costs. In contrast, job resources refer to those physical, psychological, social, or organizational aspects of the job that are either functional in achieving work goals, reduce job demands and their associated costs, or stimulate personal growth and development.

Drawing on its conceptualization of job resources, the JD-R model postulates that the enrichment of employees’ perceived job resources triggers a gain effect, which in turn fosters more proactive work attitude (Demerouti et al., 2001). This mechanism illuminates that in contexts characterized by abundant job resources—such as when employees hold a high level of trust in AI—individuals are likely to experience cumulative gains in outcomes.

Further building on its conceptualization of job demands, the JD-R model assumes that job demands can motivate employees to proactively invest their job resources into job demands (Bakker & Demerouti, 2017). This theoretical mechanism suggests that employees’ trust in AI may enable them to recognize and embrace more challenging job demands, which in turn incentivizes them to mobilize their resources and exhibit excellent outcomes.

### 2.2. Trust in AI and Innovative Behavior

Innovative behavior refers to the process in which employees identify work-related problems, develop and propose solutions or improvements, seek support for their innovative ideas, formulate concrete implementation plans, and put these plans into practice in the workplace (Scott & Bruce, 1994). This study posits that trust in AI facilitates employees’ innovative behavior, with the specific reasoning as follows: First, trust in AI reflects employees’ positive attitudes toward AI technology adoption (Höddinghaus et al., 2021) and their willingness to assume the risks associated with AI use (Choung et al., 2023), effectively representing the proactive investment of AI as a valuable job resource into work demands. Consequently, employees who trust in AI tend to leverage AI technology to improve business processes (Glikson & Woolley, 2020; W. Li et al., 2024) or address work-related problems, which can be regarded as a form of innovative behavior (Scott & Bruce, 1994). Second, when employees trust in AI, they tend to hold positive expectations of AI and focus more on the positive impacts brought about by AI. According to the JD-R model, this sense of gain may encourage employees to commit to and implement innovative activities with certain risks, thereby fostering their innovative behavior. Finally, employees’ trust in AI may facilitate the formation of a harmonious relationship with AI, which in turn is conducive to their engagement in innovative behavior. AI may impose impacts and negative effects on employees—such as a sense of crisis (Liang et al., 2022)—that may hinder innovative behavior. However, when employees trust in AI, they are more willing to accept AI (Shamim et al., 2023) and tend to establish a harmonious rather than adversarial relationship with it, thereby further promoting innovation. Extant research has also demonstrated that if the relationship between employees and AI is disharmonious, innovation is likely to decrease (Liang et al., 2022). In summary, this study proposes the following hypothesis:

**H1.**  *Trust in AI positively influences employees’ innovative behavior.*

### 2.3. Resource-Gaining Path: The Mediating Role of Job Autonomy

Job autonomy refers to the degree of freedom and discretion employees possess to coordinate resources, schedule work processes, and determine work procedures in their jobs (Hackman & Lawler, 1971). This study posits that trust in AI may enhance employees’ job autonomy through the following three mechanisms.

First, trust in AI can provide employees with additional resources for coordination, thereby helping to enhance their job autonomy. Specifically, trust itself is regarded as a positive cognitive and emotional resource (Halbesleben & Wheeler, 2015), and increased trust implies greater access to such resources. Furthermore, employees who trust in AI tend to leverage AI to accomplish work tasks, freeing up resources that were previously occupied (Jia et al., 2024). In turn, sufficient resources afford employees the space to select and coordinate resources, which strengthens their job autonomy.

Second, trust in AI helps increase employees’ discretion, thereby enhancing their job autonomy. When employees trust in AI, they tend to perceive AI as a collaborative partner (Kong et al., 2023), forming a human–AI relationship. In interpersonal relationships, employees struggle to influence others’ will or behaviors, which restricts their discretion in setting work standards and arranging work tasks. In contrast, within human–AI relationships, employees can independently select AI types, manipulate AI modes, and determine AI behaviors—experiencing relatively high levels of discretion that ultimately boost their job autonomy.

Finally, employees’ trust in AI can enhance perceived freedom to a certain extent. With technological advancement, organizations have integrated AI into the implementation of work rules and regulations (Zhang et al., 2024). Employees who trust in AI will proactively understand and adapt to AI-supported work standards, enabling them to focus more on exerting their judgment and creativity with AI assistance (Kong et al., 2024) thereby strengthening their perceived freedom. In contrast, if employees do not trust in AI, they may hold negative attitudes toward AI-based standards and become constrained by AI-driven rules (Zhang et al., 2024), which undermines their perceived freedom. Extant research has also confirmed that employees’ positive perception and reasonable response to AI can increase job autonomy (Wu & Zhang, 2024). Based on the above analysis, we propose the following hypothesis:

**H2.**  *Trust in AI positively influences job autonomy.*

A central proposition of the JD-R model is that job resources exert a positive impact on employees (Demerouti et al., 2001) and buffer the draining effects of job demands (Bakker et al., 2005), ultimately fostering employees’ positive states. This study argues that enhanced job autonomy can facilitate employees’ innovative behavior, with the specific analysis as follows: First, a high level of job autonomy means employees can fully allocate and deploy resources, applying them to the details of work processes. To a certain extent, this enables employees to identify subtle aspects of work-related problems and propose innovative solutions, thereby promoting innovative behavior (Bindl et al., 2019). Second, employees with high job autonomy benefit from a free work environment that provides them with greater room to exert their potential. This enables employees to generate innovative ideas more easily, promote and implement these ideas in the workplace and thereby foster their innovative behavior (Bindl et al., 2019; Purc & Laguna, 2019). Finally, job autonomy can generate a gain effect. The perception of abundant resources positively influences employees (Demerouti et al., 2001), motivating them to proactively implement innovative behavior that may consume resources (Černe et al., 2017; Orth & Volmer, 2017; Slåtten & Mehmetoglu, 2011). In conjunction with Hypothesis 2, we put forward the following:

**H3.**  *Trust in AI positively influences employees’ innovative behavior by enhancing job autonomy.*

### 2.4. Demand-Enabling Path: The Mediating Role of Concentration of Work-Related Flow

Concentration of work-related flow is defined as employees’ full immersion in work (Bakker, 2008), manifested by total engagement and high concentration (Mathwick & Rigdon, 2004). This flow state is only achievable when individuals perceive a balance between the challenge of a situation and their own skills to deal with this challenge to a certain extent (Csikszentmihalyi, 2000). Consistent with the JD-R model, job demands can empower employees to some extent and prompt them to actively participate in the work context (Bakker et al., 2010). Given this, we argue that trust in AI can increase employees’ concentration of work-related flow for the following reasons:

First, trust in AI can prompt employees to recognize challenging new job demands and enhance their ability to cope with such demands, which in turn helps increase their concentration of work-related flow. On the one hand, trust in AI implies employees hold positive expectations of AI (Gkinko & Elbanna, 2023). This positive perception of AI may act as a challenge stressor, motivating them to more actively embrace challenging new AI-related job demands—such as proactively exploring ways to integrate AI to improve work processes (Glikson & Woolley, 2020; W. Li et al., 2024). On the other hand, when employees trust in AI, they tend to be more proactive to a certain extent (Zhou et al., 2024) and strive to acquire AI-related knowledge and skills (Kong et al., 2024), thereby enhancing their ability to address the aforementioned job demands. At this point, employees face job demands with a certain level of challenge and possess the corresponding competence to cope with them (Csikszentmihalyi, 2000), facilitating their entry into a concentration of work-related flow (Bakker et al., 2010).

Second, trust in AI may impose new requirements on employees’ work relationships, which in turn helps enhance their concentration of work-related flow. Specifically, when employees trust in AI, they tend to recognize AI as an integral part of the work team (Glikson & Woolley, 2020) and need to properly manage the new type of work relationship that includes AI—i.e., adding a new job demand: human-AI relationship management. Meanwhile, a high level of trust in AI implies a harmonious and friendly relationship between employees and AI (McKnight & Chervany, 2001), enabling employees to more easily meet the requirements of human-AI relationship management. Consequently, they are more likely to enter a state of concentration of work-related flow.

Finally, trust in AI can free up work resources for employees to address challenging work tasks, which in turn helps improve their level of concentration of work-related flow. When employees trust in AI, they tend to rely on AI to complete tedious, repetitive, and codifiable work tasks (Jia et al., 2024). This allows them to allocate more time and energy to undertaking more challenging work tasks, which contributes to generating a higher level of concentration of work-related flow. Based on the above reasoning, we propose the following hypothesis:

**H4.**  *Trust in AI positively influences employees’ concentration of work-related flow.*

As proposed by the JD-R model, optimal outcomes are achieved when employees have abundant resources to meet highly challenging job requirements (Kwon & Kim, 2020). Concentration of work-related flow reflects a state in which employees possess sufficient ability to handle high-challenge demands. Higher levels of this flow state signify that job demands empower employees to act proactively, thereby facilitating their innovative behavior. Detailed reasoning is provided below:

First, when employees enter a state of concentration of work-related flow, they will focus their attention and engage fully in their work—activating high levels of cognitive efficiency and work motivation (Liu et al., 2023). They become immersed in reflecting on how to accomplish work tasks, which reduces the impact of external distractions, makes it easier to generate innovative ideas, and thereby helps promote innovative behavior (Hoffman & Novak, 2009). Second, under a high level of concentration of work-related flow, abundant resources are allocated to addressing challenging job demands, thereby motivating employees to engage in innovative behavior. On the one hand, employees invest abundant resources (Gerpott et al., 2022) and achieve efficient resource conversion (Taser et al., 2022), which may drive the implementation of innovative behavior. On the other hand, the fulfillment of highly challenging job demands by job resources enables employees to maintain a positive state (Bakker et al., 2005), which helps employees better generate innovative ideas, promote these ideas more actively, facilitates the generation of innovative behavior. Extant research has also confirmed that a high level of flow state can increase innovative behavior (Maqbool et al., 2019; Peng et al., 2024). Therefore, combined with Hypothesis 4, this study proposes the following hypothesis:

**H5.**  *Trust in AI positively influences employees’ innovative behavior by enhancing concentration of work-related flow.*

### 2.5. The Moderating Role of Job Complexity

Job complexity refers to the requirements for professional skills, problem-solving capabilities, psychological qualities, and other competencies needed to complete a job (Hatcher et al., 1989). Given that AI struggles to effectively accomplish non-procedural and hard-to-codify work tasks (Jia et al., 2024), the impact of trust in AI on employees may vary across different levels of job complexity. Therefore, this study argues that job complexity plays a moderating role in both the resource gain path and the demand-empowering path through which trust in AI influences innovative behavior.

Specifically, when the level of job complexity is high, the positive relationship between trust in AI and job autonomy will be weakened. First, when the level of job complexity is high, trust in AI may provide fewer resources that can be coordinated. High-complexity work tasks typically require a high level of knowledge and skills (Hatcher et al., 1989). When confronting such tasks, although employees trust AI and anticipate using it (Chi et al., 2023), AI still struggles to address these non-procedural and hard-to-codify complex tasks (Jia et al., 2024), meaning the time and energy occupied by work cannot be freed up by AI. In other words, under high job complexity, even if employees trust in AI, it is difficult for them to obtain coordinated resources, which is not conducive to enhancing job autonomy. Second, even with trust in AI, employees facing high-complexity work tasks may struggle to perceive a sense of freedom. Although AI is now widely applied in workplace settings (Höddinghaus et al., 2021), given that complex tasks are difficult to codify (Jia et al., 2024), such tasks are likely to be less constrained by AI-driven rules. Consequently, trust in AI can hardly help employees cope with AI-related rules to reduce external constraints, thereby limiting the sense of freedom perceived by employees and ultimately hindering the development of job autonomy. Therefore, this study proposes the following hypothesis:

**H6a.**  *Job complexity weakens the positive effect of trust in AI on job autonomy.*

In addition, high job complexity will attenuate the positive association between trust in AI and concentration of work-related flow. On the one hand, the empowering effect of trust in AI on job demands is weakened. High job complexity implies that employees already face highly challenging work tasks (Shaw & Gupta, 2004). In this context, even if employees trust in AI, they cannot rely on AI to complete high-complexity work tasks (Jia et al., 2024). Therefore, compared with the high inherent challenge of the work itself, the role of trust in AI in enhancing the challenge of job demands may not be significant, making it less likely for concentration of work-related flow to arise from trust in AI. On the other hand, high job complexity restricts employees’ access to resources derived from trust in AI, resulting in the new job demands recognized through trust in AI (e.g., continuous learning of AI usage) exceeding employees’ capacity to cope. In this state, employees experience a sense of incompetence and find it difficult to enter a state of concentration of work-related flow. Therefore, this study proposes the following hypothesis:

**H6b.**  *Job complexity weakens the positive effect of trust in AI on concentration of work-related flow.*

Based on Hypotheses 3 and 5, this study posits that employees’ trust in AI positively influences their innovative behavior by facilitating job autonomy and concentration of work-related flow. Furthermore, integrating Hypotheses 6a and 6b, this study argues that the mediating roles of job autonomy and concentration of work-related flow in the relationship between trust in AI and innovative behavior may also be moderated by job complexity. Specifically, when confronting high-complexity work tasks, employees are unlikely to enhance their job autonomy and concentration of work-related flow through trust in AI, thereby limiting the generation of innovative behavior. Based on the above reasoning, this study proposes the following hypotheses:

**H7a.**  *Job complexity negatively moderates the mediating effect of job autonomy in the relationship between trust in AI and innovative behavior.*

**Figure 1:**
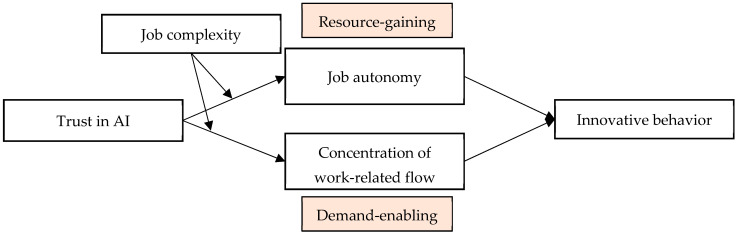
Research model.

**H7b.**  *Job complexity negatively moderates the mediating effect of concentration of work-related flow in the relationship between trust in AI and innovative behavior. Integrating all the aforementioned hypotheses, this study constructs a comprehensive theoretical framework, as illustrated in Figure 1.*

## 3. Methods

### 3.1. Participants and Procedure

The target research subjects of this study are employees who have recently been exposed to or used AI in their work. This study conducted a two-wave questionnaire survey via the Credamo platform, with a two-week interval between the two waves. To obtain sample data that meets the requirements of the research subjects, this study follows the protocols of existing literature by providing a detailed definition of AI at the outset of the questionnaire and designing a set of AI-related screening questions to verify that the participants have indeed been exposed to or utilized AI in their workplace settings (Man Tang et al., 2022). In addition, an explanation of the questionnaire survey was provided, covering the purpose of the survey, voluntary participation, and confidentiality guarantees.

The time 1 survey measuring trust in AI, job complexity, and demographic characteristics obtained 373 valid questionnaires. Through platform account matching, the time 2 survey, which inquired into job autonomy, concentration of work-related flow, and innovative behavior, was administered to the aforementioned 373 participants two weeks later, with a total of 318 questionnaires retrieved. Based on the AI-related screening questions, this study excluded participants who had no exposure to AI in their work over the past month or whose descriptions of AI technologies were clearly inconsistent with the definition of AI (e.g., the Internet, Microsoft Office). In addition, participants who completed the questionnaire carelessly (e.g., selecting the same option for all items, demonstrating a regular response pattern) or with an excessively short response time (less than 300 s) were eliminated. Ultimately, a final sample of 254 valid matched questionnaires was obtained from the 318 matched responses collected in the second wave, corresponding to an effective response rate of 79.87%. The demographic characteristics of the valid sample are as follows: the average age was 30.867 years (SD = 6.781); males accounted for 42.5%; respondents with a bachelor’s degree made up 71.3%; and the average tenure was 6.142 year (SD = 5.939).

### 3.2. Measures

All measurement items for the variables in this study were adopted from well-established scales and translated following a rigorous back-translation procedure from English to Chinese. A 5-point Likert-type scale was used for all scale items, where “1” indicates “strongly disagree” and “5” indicates “strongly agree.”

Trust in AI: Measured using a 3-item scale adapted from Höddinghaus et al. (2021) (sample item: “I feel comfortable relying on artificial intelligence”), with a Cronbach’s α of 0.726.

Job complexity: Measured via a 3-item scale developed by Zacher and Frese (2011) (sample item: “My work tasks are unusual and particularly difficult”), with a Cronbach’s α of 0.613.

Job autonomy: Measured using a 3-item scale developed by Spreitzer (1995) (sample item: “My job allows me to handle and be responsible for quite a few tasks on my own”), with a Cronbach’s α of 0.714.

Concentration of work-related flow: Measured via the absorption dimension of Bakker (2008) work-related flow scale (4 items; sample item: “When I am working, I forget everything else around me”), with a Cronbach’s α of 0.872.

Innovative behavior: Measured using a 6-item scale developed by Scott and Bruce (1994) (sample item: “I come up with creative ideas at work”), with a Cronbach’s α of 0.901.

Control Variables: Based on existing literature, employees of different ages and educational backgrounds may hold varying attitudes toward AI and other new technologies. Additionally, gender and work experience are likely to influence employees’ job demands and job resources. Therefore, this study included age, gender, educational level, and work tenure as control variables to rule out potential confounding effects (Jia et al., 2024; Malhotra & Ramalingam, 2023; Man Tang et al., 2022).

## 4. Results

### 4.1. Results of the Confirmatory Factor Analysis

Mplus was used for data processing and analysis. Harmans single-factor test was employed to check for common method bias, revealing five factors with the first factor accounting for 36.413% of the variance (≤40%), indicating no severe common method bias. Confirmatory Factor Analysis was conducted to test model fit. As presented in Table 1, the five-factor model (trust in AI, job complexity, job autonomy, concentration of work-related flow, innovative behavior) outperformed other competing models, with acceptable fit indices (χ^2^/df = 2.268, CFI = 0.921, TLI = 0.905, RMSEA = 0.071), confirming good model fit. Discriminant validity and convergent validity were further tested based on the five-factor model. All item factor loadings of the variables were greater than 0.5, all AVEs were greater than 0.5, and all CRs were greater than 0.7, indicating that the data in this study exhibits acceptable measurement validity.

### 4.2. Descriptive Statistics and Correlation Analysis

Table 2 presents the descriptive statistics and correlation analysis results of all variables in this study. Correlation analysis indicates that trust in AI is significantly and positively correlated with innovative behavior (r = 0.234, *p* < 0.01), job autonomy (r = 0.195, *p* < 0.01), and concentration of work-related flow (r = 0.265, *p* < 0.01), respectively. Additionally, innovative behavior is significantly and positively correlated with job autonomy (r = 0.598, *p* < 0.01) and concentration of work-related flow (r = 0.592, *p* < 0.01). These correlation patterns are consistent with the hypothesized relationships among the variables.

### 4.3. Hypotheses Testing

Direct effect tests were performed via Mplus, with results reported in Table 3. Trust in AI significantly and positively influences innovative behavior (B = 0.200, *p* < 0.001), supporting H1.

**Figure 2:**
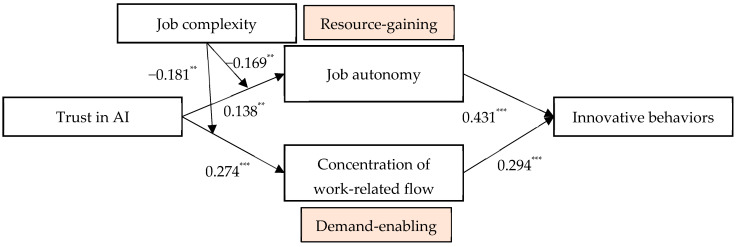
Path coefficient diagram. ** indicates *p* < 0.01, *** indicates *p* < 0.001.

Based on Figure 1 and Table 3, this study constructed a path coefficient diagram, as illustrated in Figure 2. The results indicate that trust in AI has a significantly positive effect on job autonomy (B = 0.138, *p* < 0.01) and concentration of work-related flow (B = 0.274, *p* < 0.001), thereby supporting Hypotheses 2 (H2) and 4 (H4). Furthermore, both job autonomy (B = 0.431, *p* < 0.001) and concentration of work-related flow (B = 0.294, *p* < 0.001) are significantly and positively correlated with innovative behavior. Building on these findings, this study further tested the indirect effects of trust in AI on innovative behavior through the two mediating variables.

As shown in Table 4, the indirect effect of job autonomy in the relationship between trust in AI and innovative behavior is 0.059, with a 95% confidence interval (CI) of [0.023, 0.110] that does not include zero, further supporting Hypothesis 3 (H3). Additionally, the indirect effect of concentration of work-related flow between trust in AI and innovative behavior is 0.080, with a 95% CI of [0.039, 0.146] (note: corrected the lower bound of the CI to avoid redundancy and ensure logical range) that excludes zero, providing further support for Hypothesis 5 (H5). Moreover, when considering the mediating roles of the two variables, the direct effect of trust in AI on innovative behavior (see Table 3) becomes non-significant (B = 0.048, *p* > 0.05). These results indicate that job autonomy and concentration of work-related flow exert a full mediating effect in the relationship between trust in AI and innovative behavior.

As depicted in Figure 2, the interaction term between trust in AI and job complexity significantly and negatively affects job autonomy (B = −0.169, *p* < 0.01) and concentration of work-related flow (B = −0.181, *p* < 0.01). These results provide preliminary support for Hypotheses 6a and 6b. Building on these findings, simple slope analysis was further conducted. As shown in Table 5, when job complexity is at a high level, the effect of trust in AI on job autonomy is non-significant (B = −0.031, 95% CI = [−0.182, 0.135]). In contrast, when job complexity is at a low level, trust in AI has a significantly positive effect on job autonomy (B = 0.307, 95% CI = [0.177, 0.451]), and the difference between the high and low levels of job complexity is statistically significant (B = −0.339, 95% CI = [−0.563, −0.106]). These results further support Hypothesis 6a. Similarly, when job complexity is high, the impact of trust in AI on concentration of work-related flow is non-significant (B = 0.092, 95% CI = [−0.106, 0.316]). However, when job complexity is low, trust in AI significantly and positively influences concentration of work-related flow (B = 0.455, 95% CI = [0.261, 0.670]), with a significant difference between the two levels (B = −0.362, 95% CI = [−0.660, −0.041]), thereby providing further support for Hypothesis 6b.

**Figure 3:**
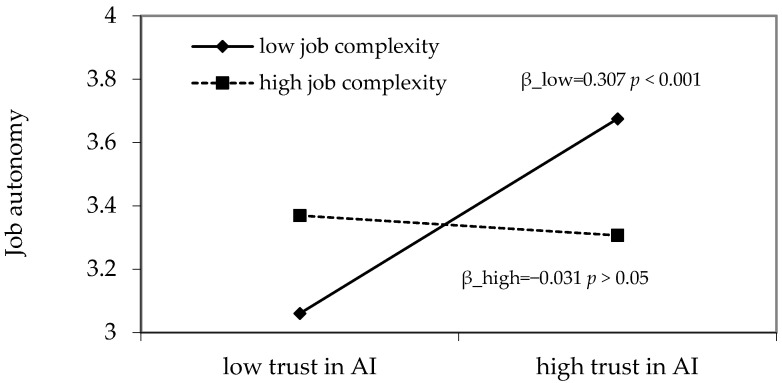
Interaction between trust in AI and job complexity on job autonomy.

**Figure 4:**
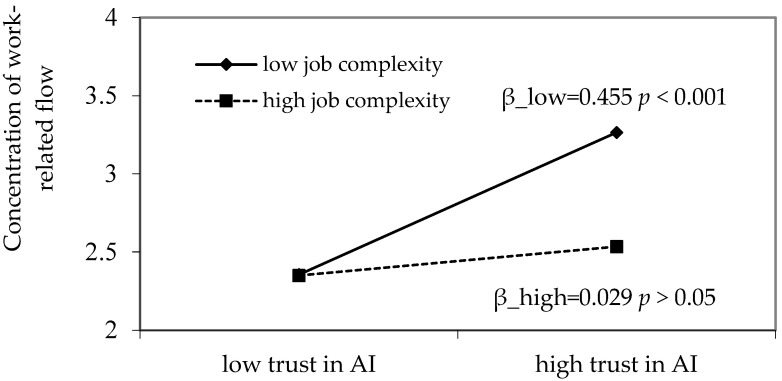
Interaction between trust in AI and job complexity on concentration of work-related flow.

In order to observe the moderating effect of work complexity more clearly, this study draws Figure 3 and Figure 4, and takes the standardized mean of work complexity plus or minus one standard deviation as the high and low groups, indicating that when work complexity is at different levels, trust in AI has an impact on job autonomy or concentration of work-related flow, respectively.

Building on the supported mediating and moderating effects, this study further tested the moderated mediation effects, with results presented in Table 6. When job complexity is at a high level, the mediating effect of job autonomy is non-significant (B = −0.013, 95% CI = [−0.083, 0.057]). In contrast, when job complexity is at a low level, the mediating effect of job autonomy is statistically significant (B = 0.132, 95% CI = [0.076, 0.218]), and the difference in the mediating effect between the two levels is significant (B = −0.146, 95% CI = [−0.272, −0.054]), thereby supporting Hypothesis 7a. Furthermore, when job complexity is high, the mediating effect of concentration of work-related flow is non-significant (B = 0.027, 95% CI = [−0.029, 0.102]). However, when job complexity is low, this mediating effect becomes significant (B = 0.134, 95% CI = [0.073, 0.238]), with a significant difference between the high and low levels (B = −0.106, 95% CI = [−0.230, −0.015]), providing support for Hypothesis 7b.

## 5. Discussion

In the context of the rapid advancement of AI—a pervasive work context confronting all employees—this study delved into the changes in employees’ job resources, job demands, and workplace behaviors when they developed trust in AI. Grounded in the JD-R model, we adopted a novel perspective that integrated individual and work-related factors, thereby preliminarily unpacked the “black box” of the mechanisms through which employees’ trust in AI influenced their innovative work behavior. The findings of this study provided empirical evidence supporting the impact of employees’ trust in AI. Specifically, we found that employees’ trust in AI not only predicted their innovative work behavior (H1) but also exerted a direct effect on their job autonomy and concentration of work-related flow (H2 and H4). Furthermore, the results revealed that employees could benefit from resource-gaining and demand-enabling effect, which supported the parallel mediating effects in the indirect relationship between employees’ trust in AI and innovative behavior (H3 and H5).

We further found that the effects of employees’ trust in AI on job autonomy, concentration of work-related flow, and subsequent innovative behavior were contingent on job complexity (Hypotheses 6a, 6b, 7a, 7b). Specifically, for employees engaged in high-complexity jobs, there were no significant differences in job autonomy, concentration of work-related flow, or innovative work behavior across different levels of trust in AI. This indicated that employees in high-complexity jobs might hardly benefit from trusting AI In contrast, for employees in low-complexity jobs, the results showed that higher levels of trust in AI predicted increased job autonomy, concentration of work-related flow, and subsequent innovative behaviors. This suggested that employees engaged in low-complexity jobs were more likely to benefit from trusting AI.

In summary, the findings clarify the multifaceted impacts of employees’ trust in AI. By revealing the parallel mediating roles of job autonomy and concentration of work-related flow, and the moderating role of job complexity, we have preliminarily unpacked the “black box” of the mechanisms underlying the relationship between trust in AI and innovative behavior, offering a new perspective for investigating the effects of employees’ trust in AI.

### 5.1. Theoretical Contributions

The development of the theoretical framework and empirical analyses in this study make several important contributions to the existing literature. First, by exploring the impact of trust in AI on innovative behavior, this study enriches the literature on the outcomes of trust in AI. Existing research on AI perceptions has predominantly focused on employees’ non-positive perceptions, such as AI awareness (Liang et al., 2022), with relatively insufficient attention paid to trust in AI—a positive perception of AI held by individuals. In relevant studies on trust in AI, this construct is often identified as a critical predictor of employees’ AI usage (Choung et al., 2023) or effective collaboration with AI (Bedué & Fritzsche, 2022). Some scholars have also found that trust in AI can positively influence AI adoption, which in turn affects employees’ personal outcomes such as well-being and productivity (Kong et al., 2023). However, existing research has overlooked the potential impact of trust in AI on other behavioral outcomes of employees. This study explores and verifies the positive relationship between trust in AI and innovative behavior, thereby enriching the literature on employees’ trust in AI and expanding the scope of outcome variables associated with trust in AI.

Second, this study constructs a theoretical framework based on the JD-R model, preliminarily unlocking the “black box” of the mechanism through which trust in AI influences innovative behavior. Most existing studies have focused on the direct effects of trust in AI on employees’ behaviors, such as AI usage (Glikson & Woolley, 2020; Leichtmann et al., 2023) and AI collaboration (Choung et al., 2023), while neglecting the underlying mechanisms through which it acts on employees. In contrast to prior research, this study draws on the JD-R model to focus on the resource-gaining path and demand-enabling path through which trust in AI impacts employees, thereby preliminarily identifying two critical mediating mechanisms: job autonomy and concentration of work-related flow. Furthermore, this study expands the application scope of the JD-R model, responding to the call for further applying the JD-R model to AI-related research topics (Liang et al., 2022).

Finally, this study extends the boundary conditions of trust in AI research. When exploring the boundary conditions of trust in AI’s effects, existing studies have only focused on individual traits such as protean career orientation (Kong et al., 2023), neglecting work-related contextual factors. Furthermore, scholars have called for future research to examine the impacts of trust in AI within specific contextual settings (Glikson & Woolley, 2020; Vanneste & Puranam, 2024). By investigating the moderating role of job complexity, this study responds to this research call, enriches the literature on the boundary conditions of trust in AI’s functioning, and provides directions for further exploring its contextual boundaries.

### 5.2. Practical Implications

First, as trust in AI facilitates employees’ innovative behavior, managers should actively promote employee trust in AI by modeling AI use in daily work to build initial trust (Xu et al., 2024), while enterprises can optimize AI systems to improve transparency and reliability (Glikson & Woolley, 2020).

Second, given that this study confirms trust in AI can enhance employees’ innovative behavior through the resource-gaining path (Demerouti et al., 2001) and the demand-enabling path (Bakker et al., 2008), management practitioners can leverage both paths to amplify the positive impact of trust in AI on innovative behavior. For the resource-gaining path, organizations should enrich AI-related resources for employees who trust in AI, thereby improving job autonomy and stimulating innovation. For the demand-enabling path, organizations can offer training to improve employees’ competence, helping them achieve higher work-related flow and promote innovation.

Third, given that this study finds the positive effects of trust in AI are attenuated when job complexity is high, management practitioners should implement more nuanced management strategies based on both job complexity and employees’ trust levels in AI to maximize its positive impacts. enterprises should avoid blindly fostering trust in AI among all employees on a large scale. Instead, they should prioritize cultivating trust in AI among employees in low-complexity positions through the aforementioned measures, enabling trust in AI to exert its positive effects and drive innovation.

### 5.3. Limitations and Future Research

First, although this study adopted a two-wave questionnaire survey and conducted common method bias tests, all variables were measured via self-report. Future research could employ multi-source data collection methods (e.g., supervisor ratings for innovative behavior, objective AI usage data), combined with qualitative methods, meta-analytic, or experimental studies, to further validate the findings of this study. Second, although this study preliminarily uncovered the mediating mechanisms through which trust in AI influences innovative behavior based on the JD-R model, concentration of work-related flow—the mediating variable in the demand path of this study—still cannot be directly regarded as job demands. Thus, the underlying mechanisms still warrant further exploration. For instance, future research could directly measure job demand variables (e.g., job stress) and examine whether trust in AI influences innovative behavior through the demand-depleting path (Bakker & Demerouti, 2017), so as to supplement the unexamined demand-depleting path in this study. Third, despite exploring the moderating role of job complexity in the relationship between trust in AI and employee outcomes, other potential boundary conditions remain underexplored. Future research may focus on the roles of additional contextual or individual factors (e.g., leaders’ preferences toward AI, the use of AI in organizational performance appraisal, etc.) to further refine the boundary conditions under which trust in AI operates. Finally, although this study enriches the literature on the outcomes of trust in AI to a certain extent, the discussion on how AI perceptions influence employees remains incomplete. Future research could further explore how employees’ negative and positive AI perceptions simultaneously exert differential impacts on individuals.

## 6. Conclusions

With the rapid advancement of AI, whether to foster employees’ trust in AI has emerged as a critical challenge that organizations and leaders who attach great importance to employee innovation must address. Focusing on the effects of employees’ trust in AI, this study finds that employees who trust AI can benefit from resource enrichment and be empowered by job demands, thereby engaging in more innovative work behavior—and this relationship is contingent on the level of job complexity. By offering a novel perspective to examine the impacts of employees’ trust in AI, this study not only informs future research to advance the understanding of trust in AI but also provides actionable insights for organizations to decide whether to cultivate employees’ trust in AI and promote employee innovation in the current AI-driven workplace.

## Figures and Tables

**Table 1:** Results of confirmatory factor analysis.

Models	χ^2^	df	χ^2^/df	CFI	TLI	RMSEA
Five-factor model: TIA, JC, JA, CWF, IB	321.994	144	2.268	0.921	0.905	0.071
Four-factor model: TIA, JC, JA + CWF, IB	453.320	146	3.105	0.865	0.841	0.091
Three-factor model: TIA + JC, JA + CWF, IB	610.011	149	4.094	0.797	0.767	0.111
Two-factor model: TIA + JC, JA + CWF + IB	767.702	151	5.051	0.731	0.695	0.127
One-factor model: TIA + JC + JA + CWF + IB	872.939	152	5.743	0.683	0.643	0.137

Note: TIA denotes trust in AI, JC denotes job complexity, JA denotes job autonomy, CWF denotes concentration of work-related flow, and IB denotes innovative behavior; “+” indicates the combination between variables.

**Table 2:** Results of descriptive statistics and correlation analysis.

Variables	Mean	SD	1	2	3	4	5	6	7	8	9
1.age	30.827	6.781	—								
2.sex	0.425	0.495	0.247 **	—							
3.education	3.142	0.551	0.034	−0.135 *	—						
4.tenure	6.142	5.939	0.845 **	0.285 **	0.012	—					
5.trust in AI	3.303	0.837	0.119	0.091	−0.151 *	0.093	—				
6.job complexity	2.907	0.791	0.289 **	0.132 *	0.012	0.339 **	0.008	—			
7.job autonomy	4.071	0.657	0.042	−0.016	0.005	−0.027	0.195 **	−0.018	—		
8.concentration of work-related flow	3.643	0.920	0.106	0.022	0.026	0.043	0.265 **	0.004	0.472 **	—	
9.innovative behavior	4.012	0.714	0.065	0.068	0.041	−0.048	0.234 **	−0.078	0.598 **	0.592 **	—

Note: N = 254; * indicates *p* < 0.05, ** indicates *p* < 0.01; the same notation applies below.

**Table 3:** Results of direct effect analysis.

Path		Path Coefficient	SE	Lower Limit of 95% CI	Upper Limit of 95% CI
Outcome Variable	Predictor Variable				
innovative behavior	Trust in AI	0.200 ***	0.055	0.097	0.314
Job autonomy	Trust in AI	0.138 **	0.048	0.045	0.236
	Trust in AI × job complexity	−0.169 **	0.057	−0.282	−0.053
Concentration of work-related flow	Trust in AI	0.274 ***	0.069	0.134	0.405
	Trust in AI × job complexity	−0.181 **	0.079	−0.330	−0.021
innovative behavior	Trust in AI	0.048	0.042	−0.034	0.132
	Job autonomy	0.431 ***	0.077	0.287	0.590
	Concentration of work-related flow	0.294 ***	0.065	0.169	0.426

Note: Regression coefficients are unstandardized coefficients; all variables were mean-centered; number of bootstrap samples = 5000; the same notation applies below. ** indicates *p* < 0.01, *** indicates *p* < 0.001.

**Table 4:** Indirect effect analysis results.

Mediators	Indirect Effect	SE	Lower Limit of 95% CI	Upper Limit of 95% CI
job autonomy	0.059	0.022	0.023	0.110
concentration of work-related flow	0.080	0.028	0.036	0.146

**Table 5:** Results of Simple slope test.

Outcome Variables	Levels of Job Complexity	Effect	SE	Lower Limit of 95% CI	Upper Limit of 95% CI
Job autonomy	High (+SD)	−0.031	0.080	−0.182	0.135
	low (+SD)	0.307	0.069	0.177	0.451
	difference	−0.339	0.115	−0.563	−0.106
Concentration of work-related flow	High (+SD)	0.092	0.108	−0.106	0.316
	low (+SD)	0.455	0.103	0.261	0.670
	difference	−0.362	0.159	−0.660	−0.041

**Table 6:** Results of Moderated Mediating Effect Bootstrap Test.

Path	Levels of Job Complexity	Indirect Effect	SE	Lower Limit of 95% CI	Upper Limit of 95% CI
Trust in AI → job autonomy → innovative behavior	High (+SD)	−0.013	0.035	−0.083	0.057
	low (+SD)	0.132	0.035	0.076	0.218
	difference	−0.146	0.054	−0.272	−0.054
Trust in AI → concentration of work-related flow → innovative behavior	High (+SD)	0.027	0.033	−0.029	0.102
	low (+SD)	0.134	0.044	0.073	0.238
	difference	−0.106	0.054	−0.230	−0.015

## Data Availability

The data and models used during the study are available from the corresponding author by request.

## References

[B1-behavsci-16-00425] Bakker A. B. (2008). The work-related flow inventory: Construction and initial validation of the WOLF. Journal of Vocational Behavior.

[B2-behavsci-16-00425] Bakker A. B., Demerouti E. (2017). Job demands-resources theory: Taking stock and looking forward. Journal of Occupational Health Psychology.

[B3-behavsci-16-00425] Bakker A. B., Demerouti E., Dollard M. F. (2008). How job demands affect partners’ experience of exhaustion: Integrating work-family conflict and crossover theory. Journal of Applied Psychology.

[B4-behavsci-16-00425] Bakker A. B., Demerouti E., Euwema M. C. (2005). Job resources buffer the impact of job demands on burnout. Journal of Occupational Health Psychology.

[B5-behavsci-16-00425] Bakker A. B., Van Veldhoven M., Xanthopoulou D. (2010). Beyond the demand-control model: Thriving on high job demands and resources. Journal of Personnel Psychology.

[B6-behavsci-16-00425] Bedué P., Fritzsche A. (2022). Can we trust AI? An empirical investigation of trust requirements and guide to successful AI adoption. Journal of Enterprise Information Management.

[B7-behavsci-16-00425] Bindl U. K., Unsworth K. L., Gibson C. B., Stride C. B. (2019). Job crafting revisited: Implications of an extended framework for active changes at work. Journal of Applied Psychology.

[B8-behavsci-16-00425] Chi O. H., Chi C. G., Gursoy D., Nunkoo R. (2023). Customers’ acceptance of artificially intelligent service robots: The influence of trust and culture. International Journal of Information Management.

[B9-behavsci-16-00425] Choung H., David P., Ross A. (2023). Trust in AI and its role in the acceptance of AI technologies. International Journal of Human–Computer Interaction.

[B10-behavsci-16-00425] Csikszentmihalyi M. (2000). Beyond boredom and anxiety.

[B11-behavsci-16-00425] Černe M., Hernaus T., Dysvik A., Škerlavaj M. (2017). The role of multilevel synergistic interplay among team mastery climate, knowledge hiding, and job characteristics in stimulating innovative work behavior. Human Resource Management Journal.

[B12-behavsci-16-00425] Demerouti E., Bakker A. B., Nachreiner F., Schaufeli W. B. (2001). The job demands-resources model of burnout. Journal of Applied Psychology.

[B13-behavsci-16-00425] Esmaeilzadeh P. (2020). The effect of the privacy policy of Health Information Exchange (HIE) on patients’ information disclosure intention. Computers & Security.

[B14-behavsci-16-00425] Filippelli S., Popescu I. A., Verteramo S., Tani M., Corvello V. (2026). Generative AI and employee well-being: Exploring the emotional, social, and cognitive impacts of adoption. Journal of Innovation & Knowledge.

[B15-behavsci-16-00425] Gerpott F. H., Rivkin W., Unger D. (2022). Stop and go, where is my flow? How and when daily aversive morning commutes are negatively related to employees’ motivational states and behavior at work. The Journal of Applied Psychology.

[B16-behavsci-16-00425] Gkinko L., Elbanna A. (2023). Designing trust: The formation of employees’ trust in conversational AI in the digital workplace. Journal of Business Research.

[B17-behavsci-16-00425] Glikson E., Woolley A. W. (2020). Human trust in artificial intelligence: Review of empirical research. Academy of Management Annals.

[B18-behavsci-16-00425] Hackman J. R., Lawler E. E. (1971). Employee reactions to job characteristics. Journal of Applied Psychology.

[B19-behavsci-16-00425] Halbesleben J. R. B., Wheeler A. R. (2015). To invest or not? The role of coworker support and trust in daily reciprocal gain spirals of helping behavior. Journal of Management.

[B20-behavsci-16-00425] Hatcher L., Ross T. L., Collins D. (1989). Prosocial behavior, job complexity, and suggestion contribution under gainsharing plans. The Journal of Applied Behavioral Science.

[B21-behavsci-16-00425] He F., Liu X., Naveed R. T., Muneer S., Singh A., Tripathi A. (2025). Trust, tools, and talk: Unlocking employee creative behavior through AI communication in financial services. Acta Psychologica.

[B22-behavsci-16-00425] Hoffman D. L., Novak T. P. (2009). Flow online: Lessons learned and future prospects. Journal of Interactive Marketing.

[B23-behavsci-16-00425] Höddinghaus M., Sondern D., Hertel G. (2021). The automation of leadership functions: Would people trust decision algorithms?. Computers in Human Behavior.

[B24-behavsci-16-00425] Jia N., Luo X., Fang Z., Liao C. (2024). When and how artificial intelligence augments employee creativity. Academy of Management Journal.

[B25-behavsci-16-00425] Johnson D., Grayson K. (2005). Cognitive and affective trust in service relationships. Journal of Business Research.

[B26-behavsci-16-00425] Kong H., Bashir M. J., Hussain S., Han Y., Bashir S. (2025). The effect of artificial intelligence trust on innovation performance: Anti-fragility and knowledge sharing mediation and identity moderation. Journal of Management and Strategy.

[B27-behavsci-16-00425] Kong H., Yin Z., Baruch Y., Yuan Y. (2023). The impact of trust in AI on career sustainability: The role of employee–AI collaboration and protean career orientation. Journal of Vocational Behavior.

[B28-behavsci-16-00425] Kong H., Yin Z., Chon K., Yuan Y., Yu J. (2024). How does artificial intelligence (AI) enhance hospitality employee innovation? The roles of exploration, AI trust, and proactive personality. Journal of Hospitality Marketing & Management.

[B29-behavsci-16-00425] Kong H., Yuan Y., Baruch Y., Bu N., Jiang X., Wang K. (2021). Influences of artificial intelligence (AI) awareness on career competency and job burnout. International Journal of Contemporary Hospitality Management.

[B30-behavsci-16-00425] Kwon K., Kim T. (2020). An integrative literature review of employee engagement and innovative behavior: Revisiting the JD-R model. Human Resource Management Review.

[B31-behavsci-16-00425] Leichtmann B., Humer C., Hinterreiter A., Streit M., Mara M. (2023). Effects of explainable artificial intelligence on trust and human behavior in a high-risk decision task. Computers in Human Behavior.

[B32-behavsci-16-00425] Li J., Bonn M. A., Ye B. H. (2019). Hotel employee’s artificial intelligence and robotics awareness and its impact on turnover intention: The moderating roles of perceived organizational support and competitive psychological climate. Tourism Management.

[B33-behavsci-16-00425] Li W., Qin X., Yam K. C., Deng H., Chen C., Dong X., Jiang L., Tang W. (2024). Embracing artificial intelligence (AI) with job crafting: Exploring trickle-down effect and employees’ outcomes. Tourism Management.

[B34-behavsci-16-00425] Liang X., Guo G., Shu L., Gong Q., Luo P. (2022). Investigating the double-edged sword effect of AI awareness on employee’s service innovative behavior. Tourism Management.

[B35-behavsci-16-00425] Liu W., Bakker A. B., Tse B. T., Van Der Linden D. (2023). Does playful work design ‘lead to’ more creativity? A diary study on the role of flow. European Journal of Work and Organizational Psychology.

[B36-behavsci-16-00425] Malhotra G., Ramalingam M. (2023). Perceived anthropomorphism and purchase intention using artificial intelligence technology: Examining the moderated effect of trust. Journal of Enterprise Information Management.

[B37-behavsci-16-00425] Man Tang P., Koopman J., McClean S. T., Zhang J. H., Li C. H., De Cremer D., Lu Y., Ng C. T. S. (2022). When conscientious employees meet intelligent machines: An integrative approach inspired by complementarity theory and role theory. Academy of Management Journal.

[B38-behavsci-16-00425] Maqbool S., Černe M., Bortoluzzi G. (2019). Micro-foundations of innovation: Employee silence, perceived time pressure, flow and innovative work behaviour. European Journal of Innovation Management.

[B39-behavsci-16-00425] Mathwick C., Rigdon E. (2004). Play, flow, and the online search experience. Journal of Consumer Research.

[B40-behavsci-16-00425] McKnight D. H., Chervany N. L. (2001). What trust means in e-commerce customer relationships: An interdisciplinary conceptual typology. International Journal of Electronic Commerce.

[B41-behavsci-16-00425] Orth M., Volmer J. (2017). Daily within-person effects of job autonomy and work engagement on innovative behaviour: The cross-level moderating role of creative self-efficacy. European Journal of Work and Organizational Psychology.

[B42-behavsci-16-00425] Peng C., Yuan G., Xie M., Zhu L., Mao Y. (2024). The impact of daily flow on employees’ daily innovative behavior: Disentangling the within-level mediation effect of job involvement and the cross-level moderation effect of person-organization fit. Current Psychology.

[B43-behavsci-16-00425] Purc E., Laguna M. (2019). Personal values and innovative behavior of employees. Frontiers in Psychology.

[B44-behavsci-16-00425] Raina K., Sharma G. D., Taheri B., Dev D., Chavriya S. (2026). Artificial intelligence-driven management: Bridging innovation, knowledge creation, and sustainable business practices. Journal of Innovation & Knowledge.

[B45-behavsci-16-00425] Rammer C., Fernández G. P., Czarnitzki D. (2022). Artificial intelligence and industrial innovation: Evidence from German firm-level data. Research Policy.

[B46-behavsci-16-00425] Scott S. G., Bruce R. A. (1994). Determinants of innovative behavior: A path model of individual innovation in the workplace. Academy of Management Journal.

[B47-behavsci-16-00425] Shamim S., Yang Y., Ul Zia N., Khan Z., Shariq S. M. (2023). Mechanisms of cognitive trust development in artificial intelligence among front line employees: An empirical examination from a developing economy. Journal of Business Research.

[B48-behavsci-16-00425] Shaw J. D., Gupta N. (2004). Job complexity, performance, and well-being: When does supplies-values fit matter?. Personnel Psychology.

[B49-behavsci-16-00425] Slåtten T., Mehmetoglu M. (2011). Antecedents and effects of engaged frontline employees: A study from the hospitality industry. Managing Service Quality.

[B50-behavsci-16-00425] Spreitzer G. M. (1995). Psychological empowerment in the workplace: Dimensions, measurement, and validation. Academy of Management Journal.

[B51-behavsci-16-00425] Taser D., Aydin E., Torgaloz A. O., Rofcanin Y. (2022). An examination of remote e-working and flow experience: The role of technostress and loneliness. Computers in Human Behavior.

[B52-behavsci-16-00425] Vanneste B. S., Puranam P. (2024). Artificial intelligence, trust, and perceptions of agency. Academy of Management Review.

[B53-behavsci-16-00425] Wu T.-J., Zhang R.-X. (2024). Exploring the impacts of intention towards human-robot collaboration on frontline hotel employees’ positive behavior: An integrative model. International Journal of Hospitality Management.

[B54-behavsci-16-00425] Xu Y., Huang Y., Wang J., Zhou D. (2024). How do employees form initial trust in artificial intelligence: Hard to explain but leaders help. Asia Pacific Journal of Human Resources.

[B55-behavsci-16-00425] Zacher H., Frese M. (2011). Maintaining a focus on opportunities at work: The interplay between age, job complexity, and the use of selection, optimization, and compensation strategies. Journal of Organizational Behavior.

[B56-behavsci-16-00425] Zeng X., Li S., Yousaf Z. (2022). Artificial intelligence adoption and digital innovation: How does digital resilience act as a mediator and training protocols as a moderator?. Sustainability.

[B57-behavsci-16-00425] Zhang Z., Liu G., Pei J., Zhang S., Liu J. (2024). Perceived algorithmic evaluation and app-workers’ service performance: The roles of flow experience and challenges of gig work. Journal of Organizational Behavior.

[B58-behavsci-16-00425] Zhou Q., Chen K., Cheng S. (2024). Bringing employee learning to AI stress research: A moderated mediation model. Technological Forecasting and Social Change.

